# Between Leisure and Pressure—Veterinarians’ Attitudes towards the Care of Competition Horses in Germany, Austria and Switzerland

**DOI:** 10.3390/ani13132126

**Published:** 2023-06-27

**Authors:** Svenja Springer, Denise Isabell Mihatsch, Herwig Grimm, Florien Jenner

**Affiliations:** 1 Department of Interdisciplinary Life Sciences, Messerli Research Institute, Unit of Ethics and Human-Animal-Studies, University of Veterinary Medicine, Vienna, Medical University of Vienna, University of Vienna, 1210 Vienna, Austria; herwig.grimm@vetmeduni.ac.at; 2 Department of Companion Animals and Horses, Equine Hospital, University of Veterinary Medicine, 1210 Vienna, Austria; 3 Department of Companion Animals and Horses, Equine Hospital, Equine Surgery Unit, University of Veterinary Medicine, 1210 Vienna, Austria; florien.jenner@vetmeduni.ac.at

**Keywords:** competition horses, veterinary care, equine veterinarians, reputation, fitness to compete, veterinary ethics, questionnaire-based survey

## Abstract

**Simple Summary:**

Veterinarians face a range of challenges when attending competition horses. Athletic goals and high expectations surrounding the performance of the horse may impact treatment decisions, and the veterinarians working at competitions may feel reputational pressure in this very public working context. Using a questionnaire, we found that a majority of German, Austrian and Swiss equine veterinarians (N = 172) agreed that competition horse owners have higher expectations than the owners of leisure horses as regards their medical services, and that the veterinarian’s reputation plays a more important role. Our data also show that owners are better informed about the diagnostics and therapies that may positively impact the care of their competition horses. Using a case vignette, we established that, on the grounds of equine welfare, the majority of respondents indicated that they were against starting a dressage horse with low-grade lameness in a competition. The respondents who indicated that they would approve a start of the dressage horse indicated that a horse with a low-grade lameness was fit enough “to compete”. We conclude that clearer definitions of phrases, such as “fit to compete”, may support veterinarians to conduct their professional responsibilities during competitions and reduce the reputational stress they experience in this working context.

**Abstract:**

Equine veterinarians face a range of challenges when attending competition horses. Athletic goals may significantly impact veterinary decision making, and the veterinarian’s work can be complicated by reputational considerations and rival opinions during an assessment of whether a horse is “fit to compete”. Using an online questionnaire, we found that the majority of German, Austrian and Swiss equine veterinarians (N = 172) surveyed agreed that the owners of competition horses are more likely than owners of leisure horses to approach them with clear treatment ideas, and that the former have higher expectations of the medical services provided. The data also show that the veterinarian’s reputation plays a more important role in the competition sphere. Using a case vignette, we established that, on the grounds of equine welfare, the majority of respondents indicated that they would decide against starting a dressage horse with low-grade lameness in a competition. Those respondents who indicated that they would approve a start of the dressage horse indicated that a horse with a low-grade lameness was fit enough “to compete”. We conclude that clearer definitions of phrases, such as “fit to compete”, may be helpful in guiding veterinarians as they discharge their professional responsibilities during competitions and reduce the reputational stress they experience in this working context.

## 1. Introduction

In recent years, ethical concerns about the use of horses for competitions have been raised in both public [1,2,3,4,5] and academic debates [6,7,8,9,10,11,12,13,14,15,16]. In particular, an incident in which the gelding “Saint Boy” was punched by a coach after refusing a jump during the modern pentathlon competition at the Tokyo Olympics 2021 caused people all over the world to voice anger about animal abuse and welfare issues in equine competitions. In an online article entitled “Saint Boy’s rebellion spurs debate about ethical treatment of horses at the Olympics—and beyond” by Coulter [3], several issues raised by this case were addressed, including the ethical treatment of horses during a competition, and reflects on a questionable feature of the modern pentathlon in which the riders draw their mount from a pool and thus meet their horses just before the show jumping competition starts [3]. The Tokyo incident and subsequent worldwide public debate did not only lead to changes to the Olympic regulations applying to the modern pentathlon [17]; it stimulated a widespread scientific general debate over the use of horses in competitions, including a through reflection on animal welfare aspects [7,8,9]. Although scientific debates over the use of horses for competitions and its ethical implications is not new [16], the recent interest in the issues raised by equestrian sport and the responsibilities of stakeholders represents a significant increase in the intensity of the discussion [9,11,12].

Veterinarians play a key role in protecting the welfare of horses in various settings. Where equine athletes are concerned, they not only provide professional medical care for the horses in general, but also have a monitoring role during competitions, where they ensure the animals are healthy and fit to participate [18]. For veterinary work during competitions, the Fédération Equestre Internationale (FEI) has laid down comprehensive veterinary regulations covering well-defined responsibilities [18]. These are often the basis of the regulations for national competitions that are applied in individual countries, such as Germany [19], Austria [20] and Switzerland [21]. The veterinary regulations include the FEI Code of Conduct for the Welfare of the Horse by considering general welfare aspects, aspects related to the horse’s fitness to compete and the humane treatment of horses [18]. In addition, specific veterinary roles in competitions, such as that of the official veterinarian, veterinary control officer and treating veterinarian, together with their responsibilities and necessary certifications, are specified [18]. These regulations provide a consistent set of animal welfare standards. They form an important basis of the veterinarian’s decision making during horse competitions, including decisions about the use of medication and horses’ permission to start, i.e., participate.

However, decision making in equine medical care can be challenging, because the goals, and hence priorities, of the animal and the owner, and the rider and/or trainer and veterinarians, may differ, complicating the treatment of the horses. Along with the potential conflicts among their duties to the horse and its human caregivers, plus the unrealistic expectations about therapeutic interventions, veterinarians working with equine athletes can face additional challenges [12]. In the highly commercialized setting of equestrian sport, human–horse relationships are often influenced by competitive ambitions. A certain amount of time, financial resources, emotional investment and equine health and fitness are required to secure an outstanding performance during competitions [11]. Added to this, the veterinarians’ economic interests, and their concerns about their reputation among horse owners and veterinary colleagues, may further increase the complexity of decision making while attending competition horses [12].

Previous publications on the issues raised by the use and veterinary care of competition horses have been based mainly on the theoretical consideration of the concerns of various stakeholders [9,12,16] using the empirical data on, for example, riders, owners, coach and FEI veterinarians [8] and judges working at competitions [10]. To the best of our knowledge, no empirical studies have specifically examined veterinarians’ attitudes toward their own work and the various issues it raises, during the care of competition horses. With this in mind, it is the aim of the present questionnaire study to gain empirical insights based on the data gathered from equine veterinarians from Germany, Austria and Switzerland. We include these three countries in the study because they are all German-speaking, have virtually identical veterinary educational programs and show parallels in the structure of their veterinary professions (e.g., a high percentage of self-employed veterinarians, together with a low percentage of corporate-owned practices). They also apply, more or less, the same regulations to horse competitions [19,20,21]. Against this background, it makes good sense to include equine veterinarians from Germany, Austria and Switzerland in order to increase the study population and, hence, the sample of the questionnaire study.

However, although the identification of the differences between the three countries studied is not the focus of this study, it is still worth emphasizing that specific socio-demographic and practice-specific aspects may be associated with veterinarians’ attitudes. Intuitively, for instance, veterinarians’ work experience, the percentage of equine athletes in their patient population and their type of practice (horses only versus mixed animal practice) may be associated with their attitudes towards the care of competition horses. Equally, the level (regional, national, international) of the horse competitions at which veterinarians are working, and the number of competitions they attend during a season, may be associated with the frequency with which they are confronted with critical situations (e.g., in which they come across unfairly prepared equipment) during their work. Using a case vignette, the present study additionally aims to examine whether the veterinarians would give a start approval for a horse with slight lameness at an international horse competition, and to what extent reasons, such as the welfare of the horse, veterinarians’ reputation, competition rules or the animal’s fitness to compete, underpin their decision.

With these points in mind, the main objective of the present study is to answer the following research questions: (i) what are veterinarians’ attitudes towards various issues that arise specifically during the care of competition horses (as opposed to leisure horses) and how are these attitudes associated with socio-demographic and practice-specific factors? (ii) How do veterinarians assess various kinds of situations that characteristically arise during horse competitions and how do these assessments vary with socio-demographic and practice-specific factors? (iii) What proportion of veterinarians would give a start approval to a horse with slight lameness at an international horse competition and what reasons would be given for that decision?

## 2. Materials and Methods

### 2.1. Study Population and Recruitment of Participants

The target group of the study were veterinarians who only or primarily worked in the field of equine medicine. Equine veterinarians were recruited in cooperation with equine associations in Germany (GPM—Gesellschaft für Pferdemedizin e.V.), Austria (VÖP—Vereinigung Österreichischer Pferdemediziner) and Switzerland (SVPM—Schweizerische Vereinigung für Pferdemedizin). A link to the online questionnaire was sent via e-mail from the respective associations to 1002 German, 320 Austrian and 327 Swiss veterinarians working mainly with equines. The survey could be completed at any time between 9 November and 9 December in 2020. Reminder e-mails were sent two weeks after it opened. Our transnational questionnaire study was reviewed and approved by the head of the Ethics Committee of the Medical University in Vienna.

It could be assumed that not all equine veterinarians were members of the relevant veterinary associations in the three countries studied. Unfortunately, no statistics exist that provide reliable data to calculate the coverage error between members of veterinary associations and the “true” number of equine veterinarians in the three countries. However, in order to try to ensure comparable study samples, we decided to recruit individuals with equine associations and accept the coverage error.

### 2.2. Survey Devlopment

The questionnaire was developed based on a literature review of relevant articles and empirical studies [7,8,9,10,11,12,15,16]. It was written in German and underwent two pre-tests. In a first step, it was sent to three veterinary specialists working in the equine hospital at Vetmeduni Vienna. In a second, an online pre-test, or trial, was conducted with eleven veterinarians: three German, five Austrian and four Swiss. Any comments received that were likely to improve the quality of the study data were considered and reflected in the final version of the questionnaire. The survey was set up using the software Alchemer^®^ (Alchemer^®^ Louisville, CO, USA).

We ensured we had the informed consent of participants by informing them before directing them to the survey that completion of the questionnaire was voluntary, that they could exit the survey at any point and that responses would be anonymous and no personal information (e-mail address, etc.) would be traceable back to them. By clicking the “next” button, the participants began the survey.

### 2.3. Survey Design and Measurements

The questionnaire had four sections (see Supplementary File S1). In it, only the first question, “Please indicate the country in which you practice primarily as an equine veterinarian”, was mandatory, with “Germany”, “Austria” and “Switzerland” provided as the answer options. Then, veterinarians were able to skip questions and navigate freely between sections. In the following paragraphs, the three sections of the questionnaire addressing the research questions listed above (i.e., sections A, B and D) are described.

Section A of the questionnaire contained twelve closed-ended questions on socio-demographic (e.g., age, gender, veterinarian’s work experience) and practice-specific factors (e.g., employment status, the percentage of active competition horses among the patients). Section B included items on the care of competition horses that formed the basis for the present study. A first sub-section of section B contained 12 statements focusing on medical aspects (e.g., “Compared with veterinary care for leisure horses, owners are better informed about possible diagnostics and therapies.”), relational and performance aspects (e.g., “Compared with veterinary care for leisure horses, the human–animal relationship is characterized primarily by performance.”) or reputational aspects (e.g., "Compared with veterinary care for leisure horses, my reputation plays a more important role.”) raised specifically by the care of competition horses and how these might differ in comparison with issues raised by the care of leisure horses. Respondents were able to indicate their level of agreement with the statements in one of eight response options ranging from 1 “strongly disagree” to 7 “strongly agree”, with 8 “I don’t know” also available. A second sub-section targeted only those veterinarians who worked at equestrian events. It included seven statements examining their assessment of various situations that might arise during their work during horse competitions (e.g., “Animal owner(s) presenting competition horses with low-grade lameness.”). Participants were able to indicate the frequency of critical situations occurring during their work by selecting one of six response options ranging from 1 “not at all” to 5 “very often”, with 6 “I don’t know” also available.

The final section of the survey—section D—included a case vignette [22] designed to identify whether the veterinarians would give a start approval for a horse with slight lameness at an international horse competition and their reasons for their decision. The case vignette stated:

*You are one of three veterinarians at an international competition. During the VetCheck on the day before the competition, you discover low-grade lameness in one of the dressage horses. Your colleagues are of the opinion that the animal is “fit for competition” according to the tournament regulations and that a start permit can be issued.*

*How would you proceed?*

Respondents could choose from the following options: “I agree with the opinion of my colleagues and give a start permission”, “I am deciding against the start of the horse” and “Other”. Where veterinarians chose the options “I agree with the opinion of my colleagues and give a start permission” or “I am deciding against the start of the horse”, four reasons were provided, each of which could be categorized as “very important reason”, “important reason”, “less important reason” or “not important at all”.

### 2.4. Data Analysis

IBM^®^ SPSS^®^ Statistics version 27.0 (IBM^®^ SPSS^®^ Statistics, Chicago, IL, USA) was used for all of the analyses. Univariate descriptive statistics were presented in tables or text. Ordinal regression analyses were conducted to identify the extent to which socio-demographic and practice-specific factors varied with veterinarians’ attitudes towards, and beliefs about, issues arising specifically during the care of competition horses (as compared with those that arose in their work with leisure horses) (Supplementary File S2). We ran 12 of these analyses, in which statements were inserted as dependent variables. The answer option “I do not know” was excluded from these analyses. The categorical variables inserted in the regression analyses were gender (1 = male; 2 = female), employment status (1 = self-employed; 2 = employed), practice type (1 = practice/clinic for horses only; 2 = mixed practice/clinic) and work at horse competitions (1 = yes; 2 = no). Veterinarians’ work experience (range: 1–41 years) and the percentage of active competition horses among the patients they had treated (range: from 1 = 1–5% to 11 = 91–100%) were included as continuous variables.

We also investigated whether socio-demographic and practice-specific factors, and the average number of horse competitions per season and percentage of active competition horses as patients, were associated with the frequency with which various situations arose during veterinary work during horse competitions (Supplementary File S3). We ran seven ordinal regression analyses in which statements were inserted as dependent variables, excluding the answer option “I do not know”. The categorical variables inserted in the regression analyses were gender (1 = male; 2 = female), employment status (1 = self-employed; 2 = employed), practice type (1 = practice/clinic for horses only; 2 = mixed practice/clinic) and level of horse competitions (1 = regional, 2 = national, 3 = international). The veterinarians’ work experience (range: 1–40 years) and number of horse competitions per season (range: 1–20) were inserted as continuous variables. The significance level was 0.05.

## 3. Results

### 3.1. Socio-Demographic and Practice-Specific Factors and Work during Horse Competitions

In total, 172 veterinarians completed the questionnaire (response rate 10.4%). Of these, 105 were German (61%), 38 Austrian (22%) and 29 Swiss (17%), and 74 were male (43.8%) and 95 female (56.2%). The mean age of the study population was 43.4 ± 10.6 years old. The mean work experience in practice was 20.76 ± 10.3 years. Furthermore, 70.9% of the participants indicated to work in a practice/clinic for horses only and 25.0% of respondents indicated to work in a mixed practice/clinic. With 78.9%, the majority of participants responded to be self-employed and 17.5% of the respondents indicated to be employed. Detailed information about the socio-demographic and practice-specific aspects, covering the whole study population and each sub-population from Germany, Austria and Switzerland, is given in Supplementary File S4.

Most respondents (81.9%) confirmed that they worked at horse competitions, with 53.2% working exclusively at the regional level and 27.3% also working at the international level (Table 1).

### 3.2. Comparing Veterinarians’ Attitudes towards the Care of Competition Horses and the Care of Leisure Horses

Twelve statements were presented that explored the respondents’ attitudes towards the care of competition horses. These were designed to highlight specific factors that might differ when attitudes to the care of leisure horses were compared (Table 2). Over half of the surveyed veterinarians indicated to agree that owners of competition horses showed a better understanding regarding necessary diagnostics and/or treatments (54.2%) and were better informed about possible diagnostics and therapies (61.1%). The statements that competition horse owners have higher expectations of the veterinarian and their medical services, and that they approach professionals more often with clear treatment ideas, were also confirmed by more than half of the respondents (55.7% and 59.0%, respectively). Although 42.4% of the respondents indicated to agree that the human–animal relationship was characterized primarily by performance, more than half (58.3%) disagreed with the statement that the owners’ emotional attachment to the horse played a less important role with competition horses than it did with leisure horses. Furthermore, 57.2% of respondents indicated to agree that, in competition horse care, their reputation played a more important role than it did with leisure horses.

### 3.3. What Explains the Difference between Veterinarians’ Attitudes towards the Care of Competition Horses and Their Attitudes to the Care of Leisure Horses?

We ran twelve ordinal regression models to understand the attitudes underpinning the answers to the twelve statements in Table 2 (see Supplementary File S2). The results indicate that respondents’ work experience, especially, is associated with their attitudes. Participants with longer work experience were more likely, where equine athletes are concerned, to agree that: the human–animal relationship is characterized primarily by performance (*p* = 0.036), and that owners are better informed about possible diagnostics and therapies (*p* = 0.005), show a greater understanding of necessary diagnostics and/or treatments (*p* = 0.013) and communicate with each other more about veterinary activities (*p* < 0.001).

Respondents who indicated to work at competitions were more likely to disagree that owners of equine athletes communicate with each other more about veterinary activities (*p* = 0.024). However, those who indicated to work at competitions or have a higher percentage of active competition horses in their patient population were more likely to agree that owners show a greater understanding of necessary diagnostics and/or treatments than veterinarians who do not work at competitions (*p* = 0.028) or have a lower percentage of active competition horses as patients (*p* = 0.008).

### 3.4. Frequency of Critical Situations during Work at Competitions

In total, seven statements were presented to the respondents working at competitions to examine the frequency of potentially critical situations during the competitions (Table 3). Between 65.2 and 82.2% of the respondents indicated that they were very little or moderately confronted by situations where they disagreed with the competition judges about the assessment of the health condition of the horse, came across unfairly prepared equipment, recognized owners who presented competition horses with low-grade lameness or observed riders and trainers using improper training methods. In addition, between 31.0 and 39.1% (i.e., approximately one third) of respondents indicated that they were never confronted with the following situations: disagreement with competition organizers over the implementation of horse inspections, observation of unfairly prepared equipment and riders who wanted to compete with their active competition horse despite inadmissible medication. Less than half of the respondents (41.1%) indicated that, often or very often, riders show compliance when the veterinarian points out a violation.

We also ran seven ordinal regression models to examine whether socio-demographic and practice-specific factors (e.g., work experience, gender and employment status), the average number of horse competitions per season at which the veterinarian works and percentage of active competition horses as patients varied with veterinarians in their assessment of various situations arising during their work at horse competitions (see Supplementary File S3). One model resulted in a significant difference: self-employed respondents were confronted less often than employed respondents by situations involving unfairly prepared equipment (e.g., gaiters, fly ears) (*p* = 0.006).

### 3.5. Veterinarians’ Start Approvals for Horses with Slight Lameness at International Competitions

Participants were presented with a case vignette. The aim was to investigate how they would deal with a situation where they discovered slight lameness in one of the dressage horses and veterinary colleagues were of the opinion that the animal was “fit for competition” according to the horse competition regulations. More than half of the respondents (57.8%) indicated that they would not give a start approval in this case. Only 18.1% would agree with the opinion of their colleagues and give the approval (Table 4).

#### 3.5.1. Relative Importance of Reasons to Agree with Colleagues and Issue a Start Approval

In total, four reasons potentially underpinning the respondents’ decision to agree with their colleagues and give a start approval were provided (Table 5). The reason that the horse showed low-grade lameness only and was “fit enough to compete” was indicated as important and very important by 40.0% and 45% of the respondents, respectively. The reason that a decision against the start approval might result in a reputational risk for the rider, and hence could have a negative effect on the veterinarian in the horse competition business, was indicated as important and very important by 36.8% and 10.5% of the respondents, respectively.

#### 3.5.2. Relative Importance of Reasons to Disagree with Colleagues and Refuse to Issue a Start Approval

Again, we offered four reasons potentially underpinning the respondents’ decision not to give the horse a start approval (Table 6). Around 80% of respondents indicated that the welfare of the horse and that a slight degree of lameness is a limitation of the animal’s fitness to compete are very important reasons, and 71.7% of the respondents indicated that the information that the competition rules do not provide any safeguards where a veterinarian decides to give a start permission as less important or not important.

## 4. Discussion

The results of the study show that surveyed equine veterinarians recognize certain advantages in the medical care of competition, rather than leisure, horses. In our survey, 54.2% and 61.1% of the respondents indicated an agreement with the statements that competition horse owners have a better understanding of necessary diagnostics and/or treatments and are better informed about the possible diagnoses and therapies, respectively. This fact undoubtedly has a positive effect on the horse’s medical care and makes the veterinarian’s work easier, as he, or she, then has less need to invest time and energy in certain persuasive efforts around the necessary veterinary interventions. Although it remains speculative, a possible explanation for this could be the owners’ awareness that only a medically well-cared-for animal can perform successfully in competitions. Correspondingly, it can be assumed that animal owners within the equestrian community are especially likely to regularly exchange information about equine medical issues and their veterinarians. Recent studies underline this regular exchange in the equestrian community, which can, for example, lead to personal recommendations for certain veterinarians. For instance, Loomans and colleagues [23] found that, of the 78 competition riders they surveyed in the Netherlands, more than half visited a particular veterinarian on the recommendation of friends or colleagues. This is in line with our finding that over 40% of veterinarians agree that owners of competition horses communicate with each other about veterinary activities more than owners of leisure horses. 

The horse owner’s use of Internet resources to find medical information, and of social media platforms to enter into exchanges with other owners, may further explain these findings. In recent studies in the field of small-animal medicine veterinarians agreed that, for example, the use of Internet resources results in a greater acceptance of diagnostics and treatments [24]. Furthermore, social media platforms, such as Facebook, offer insights into veterinary practice [25] and enable exchanges between pet owners where health advice is given in or received by Facebook groups [26]. Although these studies relate to small-animal practice, it can be assumed that the same tendencies are observable in equine practice, and indeed they might even be more pronounced among owners of competition horses, as compared with the owners of leisure horses, as our results suggest.

However, high levels of information and owner exchanges can also mean that the owners approach their veterinarians with clear treatment ideas, or have higher expectations of their veterinarians and the medical services they provide. In our study, nearly 60% of the respondents indicated an agreement with these statements indicating these aspects. Some publications have addressed the kinds of conflicts that potentially arise when horses are treated between competitions. For example, Campbell [12] noted that quality of life (over the animal’s longevity) and freedom from disease and/or pain are common goals in the treatment of companion animals, whereas the treatment of horses used for competition is often motivated by short-term performance. She noted that trainers and/or owners may have a greater interest in keeping the horse in training and returning it to competition as soon as possible, instead of focusing on its welfare [12].

These claims are only partly confirmed by our results. Although more than 40% of the respondents indicated an agreement that the human–animal relationship is characterized primarily by performance, around half (49%) indicated a disagreement that owners more frequently place performance expectations above the horse’s welfare, or that treatment regression occurs more frequently due to poor owner compliance (e.g., training too early), in comparison with the situation with leisure horses. A possible explanation for this finding is that the study participants were asked how they perceive these aspects in comparison with the situation when they are attending leisure horses. Perhaps they already experienced these kinds of challenges when caring for the latter. Leisure horses are not used in competitions, obviously. Nonetheless, challenges created by owner non-compliance (e.g., starting too early with training) or performance demands may also be quite prevalent in leisure riding, which would mean that veterinarians may not notice much of a difference between the two patient groups. However, it can also be assumed that owners are aware that training too early following a horse’s injury can significantly affect its performance in the long run, which in turn can have a negative impact on the competition.

Evaluating the human–animal relationship, we found that, although more than 40% of respondents indicated an agreement that the human–animal relationship is characterized primarily by performance, almost 60% of our respondents indicated a disagreement that the emotional attachment of owners plays a less important role. Given that the status of competition horses is often discussed in terms that convey the idea that the animal is a “sport instrument”, suggesting that it is not primarily emotional, but rather performative aspects that shape the human–animal relationship [11,12], this result came as a surprise. It suggests that the high levels of performance demanded from competition horses by their owners does not necessarily preclude an emotional attachment to the animals. As much is supported by the findings of Mueller and colleagues [27], who investigated human–horse relationships and veterinary care for geriatric and non-geriatric horses. Their results show no significant differences in the degree of human–horse attachment, even though the group of non-geriatric horses (N = 1835) included over 50% of competition horses. We strongly recommend that future studies should investigate owners’ perspectives on the status of the animal, and the relationship between performative and emotional factors, in the context of leisure and competition horses.

As regards veterinarians’ work during competitions, our results show that critical situations involving things, such as the observation of unfairly prepared equipment and disagreements with competition organizers or judges over the assessment of the horses’ health condition occur very little or moderately. Between 23.3% and 39.1% of the respondents in our study stated that these situations did not arise at all. A possible explanation for these findings is that the present regulations on animal welfare during competition are being reconsidered to date. This explanation can be further supported by the results from a focus group study with participants from equestrian sport and animal welfare research [8]. Although the participants discussed the relevant welfare issues in relation to equestrian sport, they recognized the considerable progress that has already been made. For instance, the participants highlighted the tightening and increased enforcement of regulations and rules during competitions, including the FEI ban of hyperflexion in dressage and initiatives to include “healthiest condition” awards at endurance events [8].

Although, in general, critical situations rarely occur during the respondent’s work at competitions, over 20% of our study participants indicated that situations where owners presented competition horses with low-grade lameness occurred often or very often. Lameness in competition horses is caused by various things, including animal-related factors (e.g., age and conformation of the horse), management and training methods, and training surfaces and arenas [28]. In a study of 2554 horse owners, of whom over 90% had horses competing in dressage competitions, lameness, at 33%, was the most common health problem in the dressage horse [28]. Against this background, it was not surprising that the veterinarians in our study identified lameness as being more frequent than other critical situations during competitions. Since lameness can have a considerable effect on the animal’s welfare [28], its detection and correct evaluation is an important part of the equine veterinarian’s work life and requires appropriate skills and experiences [29]. Studies [30,31] indicate that experts, such as veterinarians, are more reliable than non-experts, but also that subjective evaluation of lameness in horses is not necessarily correct [29].

During competition, the evaluation of equine lameness is of particular importance, since its presence may violate the requirement that the competition horse needs to be fit to compete [18]. The FEI Code of Conduct for the Welfare of the Horse states: “Participation in competition must be restricted to fit horses and athletes of proven competence”. In relation to this, two questions arise: first, what does “fit to compete” mean, and second, how does the subjective evaluation of veterinarians impact the assessment of a horse’s fitness to compete (or not)? This rather vague formulation of the FEI regulation, given the problems of subjectivity, can lead to a disagreement between the veterinarian and the rider/owner of a horse, as well as among the veterinary professionals working at horse competitions.

With these issues in mind, the present study also investigated whether veterinarians would be for or against the start of a horse with slight lameness when two colleagues are of the opinion that the animal is “fit to compete”. While around 18% of respondents indicated to agree with their colleague’s opinion, more than half (57.8%) were against giving a start approval in this kind of case. Among the respondents who indicated to agree to the start, 85% indicated that it was a very important/important reason that it was a low-grade lameness and that the horse was “fit enough to compete”. This contradicts the attitude of the respondents who indicated to be against the start. Here, almost 100% of the respondents indicated that a very important/important reason to not agree with the colleagues was that lameness was a limitation of the animal’s fitness to compete. As already indicated, these findings suggest that there exists a mismatch of how veterinarians interpret the requirement of “fitness to compete” which can, in turn, lead to conflicts. Since the regulations and codes aim, among other things, to provide certain safeguards for veterinarians, we suggest that important terms, such as “fitness to compete”, require clearer and more transparent definitions to avoid possible disagreements and conflicts during competition.

Undoubtedly, veterinarians have the necessary expertise to develop a clearer and more transparent definition of relevant terms, such as “fitness to compete”. However, we strongly suggest interdisciplinary reflections on such important issues, involving not only equine veterinarians but also experts in the fields of animal behavior, animal welfare, ethology, (veterinary) ethicists, etc. Such interdisciplinary approaches allow for an all-encompassing debate on the one hand and, on the other hand, they can increase the provision of scientifically sound and practice-oriented proposals of reliable consensus terminology needed in this context. In addition, we believe that the results of such interdisciplinary approaches, in combination with our study results and findings of other relevant stakeholders, can be an important basis for the further development and application of guidelines and codes of conduct published by Equine Veterinary Associations and Equine Sports Associations (e.g., FEI and national associations). A recent study has shown that many observations of elite dressage horses competing in World Cup Grand Prix Competitions contradict the FEI requirements [32]. Based on video records of 147 competitors at nine venues, the authors of the study found, in particular, the frequent occurrence of head behind vertical ≥10 degrees for ≥10 s, mouth open with separation of the teeth ≥10 s and repeated tail swishing, behaviors that should be penalized according to the FEI rules [32]. The “active” implementation of the FEI rules and requirements can be questioned on the basis of these results and points to the need to focus more on the enforcement during competitions, in addition to the further development of guidelines.

Even though our data show that over half of the respondents indicate to be against giving a start approval to a lame horse, as lameness is seen as a limitation of the animal’s “fitness to compete”, recent studies indicate a high prevalence of horses showing lameness to a certain extent and/or other pain symptoms at competitions [32,33,34,35]. A possible explanation for this discrepancy could be national differences, as the results of our study are based on equine veterinarians working in the three German-speaking countries, whereas studies focusing on the observation of horses at competitions were conducted in the UK [32,33,34,35]. However, a more likely explanation for the observed differences could be that, in our case vignette, the horse’s lameness was already recognized and reported to the survey participant, whereas veterinarians in real competitions work under difficult conditions (time pressure, observation pressure by others, etc.), which, in turn, can lead to difficulties in recognizing mild lameness or pain symptoms. Furthermore, it can be assumed that veterinarians may be much more concerned about their reputation in real-life situations than when responding to a fictional case vignette.

In this context, the case vignette produced another interesting finding about veterinarians’ concerns about their reputation. Around 50% of the respondents who indicated they would grant permission to compete recognized that excluding the rider posed reputational risks and could have negative consequences. There is no doubt that in the relevant working contexts—during as well as between competitions—veterinarians need to be especially aware of reputational issues. This is underscored by another finding of our study: that almost 60% of respondents indicated an agreement that their reputation played a more important role in the care of competition horses than it did in the care of leisure horses. A negative reputation can significantly reduce income for veterinarians when owners or riders approach their rivals or they are not hired anymore to work during competitions.

Several limitations of the present study need to be acknowledged. First, we cannot claim representativeness of the general German-speaking equine veterinarian population, since the present study relied on the convenience sampling of equine veterinarians and the response rate (10.4%) was quite low. Second, even though anonymity was guaranteed, the respondents may underreport socially undesirable attitudes or overreport more desirable behaviors, which, in turn, can lead to a social desirability bias. For instance, the results of the case vignette may suffer from this bias, which may further explain the discussed discrepancy between our findings and observational studies of horses at competitions. Third, to generate an overview of several factors that are relevant during the care of competition horses, we formulated and contextualized veterinarians’ statements with reference to comparisons with their work with leisure horses. This comparative approach may have had an impact on the veterinarians’ assessments and can make their answers appear less precise. This must be taken into account when interpreting those answers. Thus, future research should explore these two working contexts independently. Finally, we expected specific socio-demographic factors, such as level, or number, of competitions to be associated with the veterinarians’ attitudes. However, our results fail to confirm this expectation. The only variable that was found to modulate (at least some of the) attitudes was veterinarians’ working background, and even here the effect was modest. With this in mind, we recommend that future studies should further explore socio-demographic and practice-specific factors that may be associated with veterinarians’ attitudes towards the care of competition horses.

## 5. Conclusions

In comparison with the care of leisure horses, the veterinary supervision of horses during competition has advantages that equine veterinarians recognize: owners’ have a better understanding of necessary diagnostics and/or treatments and possess more information about possible diagnostics and therapies. However, our study also confirmed that owners of competition horses communicate with each other more often about veterinary activities, approach their veterinarian more often with clear treatment ideas and have higher expectations of the medical services provided. This can lead to situations that are more challenging than those arising in the course of veterinary care for leisure horses. Our case vignette suggests that equine veterinarians understand the regulatory language “fit to compete” differently. This, too, may lead to situations where there are disagreements among veterinarians during competitions. Further research into the use of terminology such as this in the context of horse sport is desirable. Such research could provide veterinarians with improved guidance as to their professional responsibilities during competitions and reduce the levels of stress connected with reputational concerns in this working context. Furthermore, with respect to the significant increase in the intensity of the public discussion of the use of horses in sports, future research should shed light on how veterinarians can be sensitized about their influential role in this public discourse.

## Figures and Tables

**Table 1:** Veterinarians working at horse competitions and level of competition.

Work at Horse Competitions	N = 171
Yes	140 (81.9)
No	28 (16.4)
Do not want to specify	3 (1.8)
**Level of horse competition**	**N = 139**
Only regional level	74 (53.2)
Up to national level	27 (19.4)
Up to international level	38 (27.3)

Counts (percent).

**Table 2:** Veterinarians’ attitudes towards the care of competition horses in comparison with their attitudes to the care of leisure horses.

Nr.	Compared with Veterinary Care for Leisure Horses…		N = 165–168
**Relational and performance aspects**			
**1**	▪ the human–animal relationship is characterized primarily by performance.	Disagreement	55 (33.3)
		Neutral	40 (24.2)
		Agreement	70 (42.4)
		**Mean ± Std.**	**4.09 ± 1.347**
**2**	▪ the emotional attachment of owners to the active competition horse plays a less important role.	Disagreement	98 (58.3)
		Neutral	33 (19.6)
		Agreement	37 (22.0)
		**Mean ± Std.**	**3.38 ± 1.442**
**3**	▪ situations occur more frequently where the performance expectations of the animal owners are placed above the horse’s welfare.	Disagreement	82 (49.1)
		Neutral	23 (13.8)
		Agreement	62 (37.1)
		**Mean ± Std.**	**3.82 ± 1.538**
**4**	▪ it is more burdensome to include interests of owners (e.g., sporting success) in veterinary decision-making processes.	Disagreement	65 (39.4)
		Neutral	27 (16.4)
		Agreement	73 (44.2)
		**Mean ± Std.**	**4.04 ± 1.611**
**Reputational and financial aspects**			
**5**	▪ my reputation plays a more important role.	Disagreement	32 (19.3)
		Neutral	39 (23.5)
		Agreement	95 (57.2)
		**Mean ± Std.**	**4.64 ± 1.526**
**6**	▪ owners of active competition horses communicate with each other more about veterinary activities.	Disagreement	58 (35.6)
		Neutral	34 (20.9)
		Agreement	71 (43.6)
		**Mean ± Std.**	**4.08 ± 1.816**
**7**	▪ financial limitations on the part of the owners are rarely relevant to the treatment decision.	Disagreement	72 (43.1)
		Neutral	29 (17.4)
		Agreement	66 (39.5)
		**Mean ± Std.**	**3.86 ± 1.51**
**Medical aspects**			
**8**	▪ owners approach me more often with clear treatment ideas.	Disagreement	41 (24.7)
		Neutral	27 (16.3)
		Agreement	98 (59.0)
		**Mean ± Std.**	**4.56 ± 1.499**
**9**	▪ owners show a greater understanding of necessary diagnostics and/or treatments.	Disagreement	48 (28.6)
		Neutral	29 (17.3)
		Agreement	91 (54.2)
		**Mean ± Std.**	**4.49 ± 1.627**
**10**	▪ treatment regression occurs more frequently due to poor owner compliance (e.g., training too early).	Disagreement	80 (48.8)
		Neutral	37 (22.6)
		Agreement	47 (28.7)
		**Mean ± Std.**	**3.64 ± 1.465**
**11**	▪ owners have higher expectations of me and my medical services.	Disagreement	51 (30.5)
		Neutral	23 (13.8)
		Agreement	93 (55.7)
		**Mean ± Std.**	**4.43 ± 1.615**
**12**	▪ owners are better informed about possible diagnostics and therapies.	Disagreement	39 (23.4)
		Neutral	26 (15.6)
		Agreement	102 (61.1)
		**Mean ± Std.**	**4.57 ± 1.530**

Counts (percent). Disagreement = 1 “strongly disagree”, 2 “disagree” and 3 “somewhat disagree”; neutral = 4 “neutral (neither agree nor disagree)”; agreement = 5 “somewhat agree”, 6 “agree”, 7 “strongly agree”.

**Table 3:** Frequency of critical situations occurring during veterinarians’ work at competitions.

Nr.			N = 124–135
**1**	In the warm-up arena, I observe riders and trainers using improper training methods.	Not at all	11 (8.1)
		Very little/moderately	111 (82.2)
		Often/very often	13 (9.6)
		**Mean ± Std.**	**2.01 ± 0.423**
**2**	Riders want to compete with their competition horses despite inadmissible medication.	Not at all	40 (31.0)
		Very little/moderately	77 (59.7)
		Often/very often	12 (9.3)
		**Mean ± Std.**	**1.78 ± 0.60**
**3**	Animal owner(s) presenting competition horses with low-grade lameness.	Not at all	9 (6.7)
		Very little/moderately	96 (71.6)
		Often/very often	29 (21.6)
		**Mean ± Std.**	**2.15 ± 0.513**
**4**	When examining the equipment, I come across unfairly prepared equipment(e.g., gaiters, fly ears).	Not at all	41 (31.1)
		Very little/moderately	86 (65.2)
		Often/very often	5 (3.8)
		**Mean ± Std.**	**1.73 ± 0.526**
**5**	When I point out a violation to riders, they show understanding.	Not at all	3 (2.4)
		Very little/moderately	70 (56.5)
		Often/very often	51 (41.1)
		**Mean ± Std.**	**2.39 ± 0.537**
**6**	Disagreements arise with competition organizers over the implementation of horseInspections.	Not at all	52 (39.1)
		Very little/moderately	71 (53.4)
		Often/very often	10 (7.5)
		**Mean ± Std.**	**1.68 ± 0.608**
**7**	There is a disagreement with the competition judges over the assessment of the health condition of the competition horse.	Not at all	31 (23.3)
		Very little/moderately	93 (69.9)
		Often/very often	9 (6.8)
		**Mean ± Std.**	**1.83 ± 0.525**

Counts (percent). 1 = not at all; 2 = very little; 3 = moderately; 4 = often; 5 = very often.

**Table 4:** Veterinarians’ decisions on whether to give a start approval for a horse with slight lameness at an international horse competition.

	N = 116
I agree with the opinion of my colleagues and give a start approval.	21 (18.1)
I decide against the start approval for the horse.	67 (57.8)
Other	28 (24.1)

Counts (percent).

**Table 5:** Relative importance of reasons to agree with colleagues and issue a start approval.

Nr.	Reasons to Agree	Relative Importance	All Countries (N = 19–20)
**1**	I do not want to act against my colleagues.	Very important	1 (5.3)
		Important	7 (36.8)
		Less important	4 (21.1)
		Not important at all	7 (36.8)
		**Mean ± Std.**	**2.89 ± 0.994**
**2**	It is a low-grade lameness and the horse is “fit enough to compete”.	Very important	9 (45.0)
		Important	8 (40.0)
		Less important	2 (10.0)
		Not important at all	1 (5.0)
		**Mean ± Std.**	**1.90 ± 1.165**
**3**	Excluding the rider carries a reputational risk and may have negative consequences for me as a horse competition veterinarian.	Very important	2 (10.5)
		Important	7 (36.8)
		Less important	9 (47.4)
		Not important at all	1 (5.3)
		**Mean ± Std.**	**3.37 ± 1.012**
**4**	I feel well-covered by the competition regulations, which simply state that the horse must be “fit enough to compete”.	Very important	2 (10.5)
		Important	9 (47.4)
		Less important	3 (15.8)
		Not important at all	4 (21.1)
		**Mean ± Std.**	**2.63 ± 1.116**

Counts (percent). 1 = very important; 2 = important; 3 = Less important; 4 = not important at all.

**Table 6:** Relative importance of reasons to refuse to issue a start approval.

Nr.	Reasons to Refuse	Relative Importance	All Countries (N = 60–67)
**1**	The welfare of the horse is my priority.	Very important	54 (80.6)
		Important	13 (19.4)
		Less important	0 (0.0)
		Not important at all	0 (0.0)
		**Mean ± Std.**	**1.19 ± 0.398**
**2**	To me, even a slight degree of lameness is a limitation of the animal’s fitness to compete in this competition.	Very important	52 (78.8)
		Important	13 (19.7)
		Less important	0 (0.0)
		Not important at all	1 (1.5)
		**Mean ± Std.**	**1.24 ± 0.528**
**3**	The horse competition rules do not provide me with any safeguards if I were to decide to enter the horse in the competition.	Very important	2 (3.3)
		Important	14 (23.3)
		Less important	27 (45.0)
		Not important at all	16 (26.7)
		**Mean ± Std.**	**3.00 ± 0.844**
**4**	I would like to expose grievances without any exception at internationalCompetitions.	Very important	19 (29.2)
		Important	25 (38.5)
		Less important	10 (15.4)
		Not important at all	11 (16.9)
		**Mean ± Std.**	**2.20 ± 1.049**

Counts (percent). 1 = very important; 2 = important; 3 = less important; 4 = not important at all.

## Data Availability

All relevant data are presented in the paper and its Supplementary Materials.

## Supplementary Materials

The following supporting information can be downloaded at: https://www.mdpi.com/article/10.3390/ani13132126/s1, File S1: Questionnaire in English, File S2: Ordinal regression analyses of socio-demographic and practice-specific factors on attitudes towards the care of competition horses in comparison to leisure horses; File S3: Ordinal regression analyses of socio-demographic and practice-specific factors on frequency of critical situations occurring during veterinarians work at competitions; File S4: Socio-demographic and practice-specific aspects for the whole study population and each sub-population from Germany, Austria and Switzerland.
