# Supplementary material for: Between Leisure and Pressure—Veterinarians’ Attitudes towards the Care of Competition Horses in Germany, Austria and Switzerland
Source: Animals (Basel). 2023 Jun 27;13(13):2126. doi: 10.3390/ani13132126 (PMC10339975; doi:10.3390/ani13132126)
Supplement: Supplementary file 1 [file animals-13-02126-s001.zip › III_Supp_3_Ordinal_Regression_Analyses.pdf]

Supplementary File 3:

Ordinal regression analyses of socio-demographic and practice-specific factors on frequency of critical situations occurring during veterinarians work at competitions

**Model 1: In the warm-up arena, I observe riders and trainers using improper training methods.** (χ^2 (7)=11.492, P=0.119)					
			Hypothesis Test		
	B	Std. Error	Wald Chi-Square	df	Sig.
Gender *(ref. cat.: female)*	-,268	,3970	,456	1	,499
Employment type *(ref.cat.: employed)*	,923	,5637	2,678	1	,102
Practice type *(ref. cat.: mixed practice/clinic)*	,090	,4014	,051	1	,822
Level of horse competition (regional) *(ref.cat.: international)*	,440	,4536	,939	1	,332
Level of horse competition (national) *(ref.cat.: international)*	-,705	,5577	1,598	1	,206
Years of working experience	,019	,0233	,686	1	,407
number of horse competitions per season	-,036	,0483	,556	1	,456
**Model 2: Riders want to compete with their competition horses despite inadmissible medication.** (χ^2 (7)=1.306, P=0.988)					
Gender *(ref. cat.: female)*	-,024	,3934	,004	1	,951
Employment type *(ref.cat.: employed)*	-,207	,5420	,146	1	,703
Practice type *(ref. cat.: mixed practice/clinic)*	,181	,3877	,217	1	,641

Level of horse competition (regional) *(ref.cat.: international)*	,390	,4448	,770	1	,380
Level of horse competition (national) *(ref.cat.: international)*	,143	,5171	,077	1	,782
Years of working experience	,009	,0219	,180	1	,671
number of horse competitions per season	-,009	,0474	,037	1	,848
**Model 3: Animal owner(s) presenting competition horses with low-grade lameness.** (χ^2 (7)=10.534, P=0.160)					
Gender *(ref. cat.: female)*	-,418	,3780	1,220	1	,269
Employment type *(ref.cat.: employed)*	,964	,5236	3,391	1	,066
Practice type *(ref. cat.: mixed practice/clinic)*	,671	,3839	3,055	1	,080
Level of horse competition (regional) *(ref.cat.: international)*	-,486	,4425	1,206	1	,272
Level of horse competition (national) *(ref.cat.: international)*	,042	,5124	,007	1	,934
Years of working experience	-,030	,0221	1,839	1	,175
number of horse competitions per season	-,011	,0480	,056	1	,812
**Model 4: When examining the equipment, I come across unfairly prepared equipment (e.g. gaiters, fly ears).** (χ^2 (7)=15.869, P=0.026)					
Gender *(ref. cat.: female)*	,408	,4027	1,026	1	,311
Employment type *(ref.cat.: employed)*	-1,633	,5969	7,484	1	,006

Practice type *(ref. cat.: mixed practice/clinic)*	,008	,4105	,000	1	,984
Level of horse competition (regional) *(ref.cat.: international)*	-,729	,4957	2,162	1	,141
Level of horse competition (national) *(ref.cat.: international)*	-,826	,5691	2,106	1	,147
Years of working experience	,031	,0236	1,738	1	,187
number of horse competitions per season	,055	,0501	1,189	1	,275
**Model 5: When I point out a violation to riders, they show understanding.** (χ^2 (7)=4.069, P=0.772)					
Gender *(ref. cat.: female)*	,067	,3873	,030	1	,863
Employment type *(ref.cat.: employed)*	,751	,5587	1,807	1	,179
Practice type *(ref. cat.: mixed practice/clinic)*	-,417	,4001	1,087	1	,297
Level of horse competition (regional) *(ref.cat.: international)*	-,393	,4401	,799	1	,371
Level of horse competition (national) *(ref.cat.: international)*	-,183	,5206	,124	1	,725
Years of working experience	-,007	,0228	,099	1	,753
number of horse competitions per season	,012	,0472	,061	1	,804
**Model 6: Disagreements arise with competition organizers over the implementation of horse inspections.** (χ^2 (7)=8.548, P=0.287)					
Gender *(ref. cat.: female)*	-,712	,3908	3,322	1	,068

Employment type *(ref.cat.: employed)*	,138	,5598	,061	1	,806
Practice type *(ref. cat.: mixed practice/clinic)*	,548	,3944	1,928	1	,165
Level of horse competition (regional) *(ref.cat.: international)*	-,554	,4701	1,387	1	,239
Level of horse competition (national) *(ref.cat.: international)*	-,111	,5313	,044	1	,834
Years of working experience	,036	,0227	2,497	1	,114
number of horse competitions per season	-,070	,0491	2,005	1	,157
**Model 7: There is a disagreement with the competition judges over the assessment of the health condition of the competition horse.** (χ^2 (2)=2.432, P=0.902)					
Gender *(ref. cat.: female)*	-,121	,3885	,097	1	,756
Employment type *(ref.cat.: employed)*	-,109	,5582	,038	1	,845
Practice type *(ref. cat.: mixed practice/clinic)*	,301	,3899	,596	1	,440
Level of horse competition (regional) *(ref.cat.: international)*	-,452	,4499	1,011	1	,315
Level of horse competition (national) *(ref.cat.: international)*	-,100	,5280	,036	1	,851
Years of working experience	,018	,0230	,589	1	,443
number of horse competitions per season	-,030	,0457	,421	1	,516
