# Supplementary material for: Between Leisure and Pressure—Veterinarians’ Attitudes towards the Care of Competition Horses in Germany, Austria and Switzerland
Source: Animals (Basel). 2023 Jun 27;13(13):2126. doi: 10.3390/ani13132126 (PMC10339975; doi:10.3390/ani13132126)
Supplement: Supplementary file 1 [file animals-13-02126-s001.zip › II_Supp_2_Ordinal_Regression_Analyses.pdf]

Supplementary File 2:
Ordinal regression analyses of socio-demographic and practice-specific factors on attitudes towards the care of competition horses in comparison to leisure horses

**Model 1: Compared with veterinary care for leisure horses, financial limitations on the part of the owners are rarely relevant to the treatment decision.** (χ^2 (6)=3.328, P=0.767)					
			Hypothesis Test		
	B	Std. Error	Wald Chi-Square	df	Sig.
Gender *(ref. cat.: female)*	,071	,3258	,047	1	,829
Employment type *(ref.cat.: employed)*	,316	,4410	,513	1	,474
Years of working experience	,010	,0181	,326	1	,568
Work on competitions *ref.cat.: no)*	-,228	,4005	,325	1	,568
Practice type *(ref. cat.: mixed practice/clinic)*	,171	,3517	,237	1	,626
Percentage of active used competition horses among patients treated	,060	,0660	,833	1	,362
**Model 2: Compared with veterinary care for leisure horses, the human-animal relationship is characterized primarily by performance**. (χ^2 (6)=15.521, P=0.017)					
Gender *(ref. cat.: female)*	,433	,3292	1,733	1	,188
Employment type *(ref.cat.: employed)*	,448	,4593	,953	1	,329
Years of working experience	,040	,0190	4,383	1	,036
Work on competitions *ref.cat.: no)*	,259	,4072	,405	1	,525
Practice type *(ref. cat.: mixed practice/clinic)*	,263	,3595	,535	1	,464

Percentage of active used competition horses among patients treated	,042	,0688	,376	1	,540
**Model 3: Compared with veterinary care for leisure horses, the emotional attachment of owners to the active competition horse plays a less important role.** (χ^2 (6)=5.269, P=0.510)					
Gender *(ref. cat.: female)*	,102	,3288	,097	1	,756
Employment type *(ref.cat.: employed)*	-,167	,4343	,148	1	,700
Years of working experience	,028	,0183	2,307	1	,129
Work on competitions *ref.cat.: no)*	,099	,4137	,057	1	,811
Practice type *(ref. cat.: mixed practice/clinic)*	,097	,3592	,073	1	,788
Percentage of active used competition horses among patients treated	-,084	,0674	1,561	1	,212
**Model 4: Compared with veterinary care for leisure horses, owners are better informed about possible diagnostics and therapies.** (χ^2 (6)=12.755, P=0.047)					
Gender *(ref. cat.: female)*	-,592	,3321	3,174	1	,075
Employment type *(ref.cat.: employed)*	-,172	,4391	,154	1	,695
Years of working experience	,052	,0185	7,830	1	,005
Work on competitions *ref.cat.: no)*	-,268	,4048	,439	1	,508
Practice type *(ref. cat.: mixed practice/clinic)*	,052	,0185	7,830	1	,005
Percentage of active used competition horses among patients treated	,130	,0710	3,356	1	,067

**Model 5: Compared with veterinary care for leisure horses, owners have higher expectations of me and my medical services.** (χ^2 (6)=7.694, P=0.261)					
Gender *(ref. cat.: female)*	-,153	,3363	,206	1	,650
Employment type *(ref.cat.: employed)*	,860	,4430	3,766	1	,052
Years of working experience	,016	,0187	,727	1	,394
Work on competitions *ref.cat.: no)*	,210	,3929	,285	1	,594
Practice type *(ref. cat.: mixed practice/clinic)*	,053	,3599	,021	1	,884
Percentage of active used competition horses among patients treated	,005	,0679	,005	1	,943
**Model 6: Compared with veterinary care for leisure horses, treatment regression occurs more frequently due to poor owner compliance (e.g. training too early).** (χ^2 (6)=7.837, P=0.250)					
Gender *(ref. cat.: female)*	,246	,3384	,527	1	,468
Employment type *(ref.cat.: employed)*	,228	,4503	,256	1	,613
Years of working experience	,024	,0183	1,717	1	,190
Work on competitions *ref.cat.: no)*	,229	,4425	,269	1	,604
Practice type *(ref. cat.: mixed practice/clinic)*	,413	,3636	1,287	1	,257
Percentage of active used competition horses among patients treated	-,058	,0689	,718	1	,397
**Model 7: Compared with veterinary care for leisure horses, my reputation plays a more important role.** (χ^2 (6)=12.681, P=0.048)					
Gender *(ref. cat.: female)*	,439	,3400	1,669	1	,196

Employment type *(ref.cat.: employed)*	,560	,4415	1,611	1	,204
Years of working experience	,027	,0186	2,045	1	,153
Work on competitions *ref.cat.: no)*	-,316	,4017	,619	1	,432
Practice type *(ref. cat.: mixed practice/clinic)*	,058	,3593	,026	1	,871
Percentage of active used competition horses among patients treated	,021	,0700	,092	1	,761
**Model 8: Compared with veterinary care for leisure horses, owners approach me more often with clear treatment ideas.** (χ^2 (2)=2.432, P=0.876)					
Gender *(ref. cat.: female)*	-,211	,3306	,407	1	,524
Employment type *(ref.cat.: employed)*	,157	,4521	,120	1	,729
Years of working experience	,009	,0187	,240	1	,624
Work on competitions *ref.cat.: no)*	,322	,4110	,615	1	,433
Practice type *(ref. cat.: mixed practice/clinic)*	,009	,0187	,240	1	,624
Percentage of active used competition horses among patients treated	-,072	,0670	1,151	1	,283
**Model 9: Compared with veterinary care for leisure horses, owners show greater understanding regarding necessary diagnostics and/or treatments.** (χ^2 (6)=16.559, P=0.011)					
Gender *(ref. cat.: female)*	,028	,3237	,008	1	,930
Gender *(ref. cat.: female)*	-,826	,4518	3,339	1	,068
Employment type *(ref.cat.: employed)*	,046	,0185	6,192	1	,013

Years of working experience	-,904	,4103	4,854	1	,028
Work on competitions *ref.cat.: no)*	-,499	,3636	1,885	1	,170
Practice type *(ref. cat.: mixed practice/clinic)*	,183	,0688	7,076	1	,008
**Model 10: Compared with veterinary care for leisure horses, it is more burdensome to include interests of owners (e.g. sporting success) in veterinary decision-making processes.** (χ^2 (6)=4.166, P=0.654)					
Gender *(ref. cat.: female)*	-,245	,3345	,537	1	,464
Employment type *(ref.cat.: employed)*	,385	,4689	,674	1	,412
Years of working experience	,008	,0184	,167	1	,683
Work on competitions *ref.cat.: no)*	-,026	,4084	,004	1	,949
Practice type *(ref. cat.: mixed practice/clinic)*	,617	,3619	2,903	1	,088
Percentage of active used competition horses among patients treated	-,025	,0692	,133	1	,716
**Model 11: Compared with veterinary care for leisure horses, owners of active competition horses communicate with each other more about veterinary activities.** (χ^2 (6)=22.355, P=0.001)					
Gender *(ref. cat.: female)*	-,495	,3352	2,180	1	,140
Employment type *(ref.cat.: employed)*	-,141	,4342	,105	1	,746
Years of working experience	,069	,0188	13,526	1	,000
Work on competitions *ref.cat.: no)*	-,857	,3808	5,067	1	,024
Practice type *(ref. cat.: mixed practice/clinic)*	,521	,3660	2,024	1	,155

Percentage of active used competition horses among patients treated	,069	,0683	1,025	1	,311
**Model 12: Compared with veterinary care for leisure horses, situations occur more frequently in which the performance expectations of the animal owners are placed above the horse's welfare** (χ^2 (6)=5.101, P=0.531)					
Gender *(ref. cat.: female)*	,358	,3388	1,118	1	,290
Employment type *(ref.cat.: employed)*	,786	,4708	2,790	1	,095
Years of working experience	-,009	,0184	,234	1	,629
Work on competitions *ref.cat.: no)*	,193	,4488	,184	1	,668
Practice type *(ref. cat.: mixed practice/clinic)*	,060	,3531	,029	1	,865
Percentage of active used competition horses among patients treated	-,024	,0685	,118	1	,731
