# Supplementary material for: Between Leisure and Pressure—Veterinarians’ Attitudes towards the Care of Competition Horses in Germany, Austria and Switzerland
Source: Animals (Basel). 2023 Jun 27;13(13):2126. doi: 10.3390/ani13132126 (PMC10339975; doi:10.3390/ani13132126)
Supplement: Supplementary file 1 [file animals-13-02126-s001.zip › IV_Supp_4_Sociodemographic and practice-specific aspects.pdf]

Supplementary File 4:

Socio-demographic and practice-specific aspects for the whole study population and each sub-population from Germany, Austria and Switzerland

	All countries (N=138-172)	Germany (n=92-105)	Austria (n=23-38)	Switzerland (n=23-29)
**GENDER**				
Male	74 (43.8)	53 (51.0)	10 (27.8)	11 (37.9)
Female	95 (56.2)	51 (49.0)	26 (72.2)	18 (62.1)
**AGE (in years)**				
26-39	46 (28.2)	26 (25.8)	8 (22.2)	12 (46.2
40-49	44 (27.0)	2423.8)	14 (38.9)	6 (23.1)
50-59	54 (33.1)	39 (38.6)	11 (30.6)	4 (15.4)
≥ 60	19 (11.7)	12 (11.9)	3 (8.3)	2 (15.4)
**WORK EXPERIENCE (in years)**				
1-5	12 (7.0)	8 (7.6)	4 (10.5)	0 (0.0)
6-10	24 (14.0)	11 (10.5)	4 (10.5)	9 (32.1)
11-20	50 (29.2)	25 (23.8)	15 (39.5)	10 (35.7)
21-30	57 (33.3)	44 (41.9)	9 (23.7)	4 (14.3)
<30	28 (16.4)	17 (16.2)	6 (15.8)	5 (17.9)
**PRACTICE TYPE**				
Horse practice/clinic	122 (70.9)	80 (76.2)	23 (60.5)	19 (70.4)
Mixed practice/clinic	43 (25.0)	20 (19.0)	15 (39.5)	8 (29.6)
Other	7 (4.1)	5 (4.8)	0 (0.0)	0 (0.0)
**EMPLOYMENT STATUS**				
Self-employed	135 (78.9)	79 (76.0)	37 (97.4)	19 (65.5)
Employed	30 (17.5)	22 (21.1)	1 (2.6)	7 (24.1)
Other (retired and unspecified)	6 (3.5)	3 (2.9)	0 (0.0)	3 (10.3)
**NUMBER OF COLLEAGUES (including respondent)**				
1	78 (47.9)	43 (42.2)	22 (64.7)	13 (48.1)
2	31 (19.0)	14 (13.7)	6 (17.6)	11 (40.7)
3	18 (11.0)	13 (12.7)	5 (13.2)	0 (0.0)
> 3	36 (22.1)	32 (31.3)	1 (2.9)	3 (11.1)
**WORKING IN OTHER VETREINARY FIELDS BESIDES EQUINE PRACTICE**				
Yes	432 (66.8)	34 (32.7)	18 (47.4)	10 (34.5)
No	215 (33.2)	70 (67.3)	20 (52.6)	19 (65.5)
**PERCENTAGE OF HORSES AS PATIENTS**				
100 %	78 (45.3)	50 (47.6)	14 (36.8)	14 (48.3)
80-99%	59 (34.3)	40 (38.1)	9 (23.7)	10 (34.5)
50-79%	16 (9.3)	9 (8.6)	5 (13.2)	2 (6.9)
=/>80%	19 (11.0)	6 (5.7)	10 (26.3)	3 (10.3)
**PERCENTAGE OF ACTIVE SHOW HORSES AS PATIENTS**				
1-30%	59 (35.5)	33 (32.7)	17 (45.9)	9 (32.1)
31-60%	73 (44.0)	49 (48.5)	14 (37.8)	10 (35.7)
> 60%	34 (20.5)	19 (18.8)	6 (16.2)	9 (32.1)
**WORK AT HORSE SHOWS**				
Yes	140 (81.9)	92 (87.6)	25 (65.8)	23 (82.1)
No	28 (16.4)	13 (12.4)	12 (31.6)	3 (10.7)
Do not want to specify	3 (1.8)	0 (0.0)	1 (2.6)	2 (7.1)
**VETERINARIANS' LEVEL OF HORSE SHOWS**				
regional level	123 (88.5)	85 (62.0)	17 (42.5)	21 (51.2)
national level	57 (41.0)	30 (21.9)	12 (30.0)	15 (36.6)
international level	38 (27.3)	22 (16.1)	11 (27.5)	5 (12.2)
**AVARAGE NUMBER OF TOURNAMENTS PER SEASON**				
1-2	38 (27.5)	21 (22.8)	11 (47.8)	6 (26.1)
3-4	39 (28.3)	28 (30.4)	5 (21.7)	6 (26.1)
5-9	37 (26.8)	28 (30.4)	4 (17.4)	5 (21.7)
=/> 10	24 (17.4)	15 (16.3)	3 (13.0)	6 (26.1)

Counts (per cent)
