# Supplementary material for: Between Leisure and Pressure—Veterinarians’ Attitudes towards the Care of Competition Horses in Germany, Austria and Switzerland
Source: Animals (Basel). 2023 Jun 27;13(13):2126. doi: 10.3390/ani13132126 (PMC10339975; doi:10.3390/ani13132126)
Supplement: Supplementary file 1 [file animals-13-02126-s001.zip › I_Supp_1_Questionnaire_ENG.pdf]

"Higher, faster, broader – care for active show horses"

An online questionnaire survey on the moral challenges and medical aspects of the care for active show horses in Austria, Germany and der Switzerland

Introduction and background

Thank you very much for your participation in this questionnaire survey. The aim of this survey is to **elucidate the attitudes of horse veterinarians** from Austria, Germany and Switzerland regarding **care for show horses and to examine how these attitudes influence clinical decision processes.**
The questionnaire has four sections**. Answering the questions** will take approximately 20 minutes of your time. **Your answers will make a valuable contribution to a better understanding of ethically challenging situations and the way of dealing with them in competitive equestrian sports. The results will be published in international and veterinary journals.**

The study is carried out by the University of Veterinary Medicine, Vienna (Messerli Research Institute and University Clinic for Horses). The questionnaire was proved by the Ethics Committee of the Medical University of Vienna.

Answering the questions of this questionnaire is **voluntary.** You can **leave** the survey **at any time. Your answers are anonymous and not connected to any information that would indicate your identity.**

Thank you very much in advance for your contribution.

Please indicate in which country you are primarily working as a practising horse veterinarian: (mandatory question)

- Germany
- Austria
- Switzerland

Block A: Socio-demographic und practice-specific questions

For a start, we ask you to provide some demographic information as well as information on your professional career and your work environment.

1. *Please indicate your age in years. (drop-down list 20 – 100 years, I do not want to specify)*

_____ years

2. *Please indicate your gender.*

- male
- female
- non-binary
- I do not want to specify

3. *What is your current employment status? Please tick all that apply. (multiple choice, mandatory question because of programming)*

- Self-employed, full time
- Self-employed, part time
- Employed, full time
- Employed, part time
- Retired, but still working

- Retired
- Other
- I do not want to specify

4. *How many years have you been working as a horse veterinarian? (drop-down list 0– 80 years)*
   How many years did you work as a horse veterinarian?

   _____ years

(when "retired" is chosen, all questions and items in the following will be in past tense)

5. *Where do you currently work as a horse veterinarian? Please tick all that apply. (multiple choice)*
   Where did you last work as a horse veterinarian?

- Private out-patient horse practice
- Private out-patient mixed practice (e.g. small animals or livestock)
- Corporate out-patient horse practice (e.g. AniCura, Evidensia)
- Corporate out-patient mixed practice (e.g. AniCura, Evidensia)
- Private horse clinic
- Private mixed clinic (e.g. small animals or livestock)
- Corporate in-patient horse clinic (z.B. AniCura, Evidensia)
- University clinic
- Other

6. *In which region(s) do you currently work as a horse veterinarian? Please tick all that apply. (multiple choice)*
   In which region(s) did you work as a horse veterinarian? Please tick all that apply.

Austria:

- Western Austria (Vorarlberg, Tyrol, Salzburg, Upper Austria)
- Eastern Austria (Lower Austria, Vienna, Burgenland)
- Southern Austria (Carinthia, Styria)
- I do not want to specify

Germany:

- Baden-Wuerttemberg
- Bavaria
- Berlin
- Brandenburg
- Bremen
- Hamburg
- Hesse
- Mecklenburg-Western Pomerania
- Lower Saxony
- North Rhine-Westphalia
- Rhineland-Palatinate
- Saarland

- Saxony
- Saxony-Anhalt
- Schleswig-Holstein
- Thuringia
- I do not want to specify

Switzerland:

- Aargau
- Appenzell Outer-Rhodes
- Appenzell Inner-Rhodes
- Basel
- Bern
- Fribourg
- Geneva
- Glarus
- Grisons
- Jura
- Lucerne
- Neuchâtel
- Nidwalden
- Obwalden
- St. Gallen
- Schaffhausen
- Schwyz
- Solothurn
- Thurgau
- Ticino
- Uri
- Vaud
- Valais
- Zug
- Zurich
- I do not want to specify

7. *How many horse veterinarians work in your practice/clinic (you included)? (drop-down list 1 to 15 horse veterinarians)*
   How many horse veterinarians worked in your practice/clinic (you included)?

___________ horse veterinarians
In case there is a second work place: ___________ horse veterinarians
In case there is a third work place: ___________ horse veterinarians

8. *Do you work in other fields, besides your work as a horse veterinarian?*
   Did you work in other fields, besides your work as a horse veterinarian?

- Yes
- No

A. *Please indicate:*

- Small animals
- Livestock
- Lab animals
- Authority
- Food hygiene and food safety
- Zoo animals
- Other

9. *Do you have any of the following additional qualifications, besides your degree in veterinary medicine? Please tick all that apply.*

- Physiotherapy
- Homoeopathy
- Osteopathy
- Acupuncture
- Chiropractic
- Phytotherapy
- Complementary Medicine
- TCM (Traditional Chinese Medicine)
- Animal husbandry, animal welfare and behavioural medicine
- I do not have any of the above-mentioned additional qualifications.
- Other

10. *How many of your patients are horses?*
How many of your patients were horses?

- 100 %
- 90–99 %
- 80–89 %
- 70–79 %
- 60–69 %
- 50–59 %
- 40–49 %
- 30–39 %
- 20–29 %
- 10–19%
- 1–9 %
- I don’t know

11. *How many of the horses you treat are actively used show horses? (drop-down 1-100% + I do not treat active show horses + I don’t know)*
How many of the horses you treated were actively used show horses?

- 1–5%
- 6–10%

- 11–20%
- 21–30%
- 31–40%
- 41–50%
- 51–60%
- 61–70%
- 71–80%
- 81–90%
- 91–100%
- I do not treat active show horses.
  I did not treat active show horses.
- I don't know

12. *In which sports are the active show horses you treat used? Please tick all that apply. (multiple choice) (logical question: only if active show horses are treated)*
    In which sports were the active show horses you treated used? Please tick all that apply.

- Dressage
- Show jumping
- Eventing
- Distance
- Driving
- Reining
- Vaulting
- Horse ball
- Icelandic equitation
- Mounted games
- Mounted orienteering
- Polo
- Riding sidesaddle
- Tetrathlon
- Western riding
- Working equitation
- Harness racing
- Gallop racing
- I don't know

SUBSECTION: Veterinary work at horse shows

13. *Do you as a horse veterinarian work at horse shows?*
    Did you as a horse veterinarian work at horse shows?

- Yes
- No
- I do not want to specify

   a. *What kind of shows do you work at? Please tick all that apply. (Logical question, only if "yes" was chosen at the previous question)*
      What kind of shows did you work at? Please tick all that apply.

- Regional
- National
- International

b. *On average, how many shows do you work at in one season?*
(Due to the COVID-19 pandemic, please indicate the average number on the basis of the shows you worked at in 2019.)
On average, how many shows did you work at in one season?

- 1
- 2
- 3
- 4
- 5
- 6
- …. up to 100

Block B: Care for active show horses

Factors regarding patients, animal keepers and the professional environment when caring for active show horses

1. *The second part focuses on factors regarding patients, animal keepers and the professional environment of horse veterinarians when they* ***care for active show horses.*** *We would like to know to what extent you agree with the following statements.*

1 = strongly disagree; 2 = disagree; 3 = somewhat disagree; 4 = neutral (neither agree nor disagree); 5 = somewhat agree; 6 = agree, 7 = strongly agree and 8 = I don't know

	**Compared with veterinary care for leisure horses…**	1	2	3	4	5	6	7	8
1.	• financial limitations on the part of the owners are rarely relevant to the treatment decision.								
2.	• the human-animal relationship is characterized primarily by performance.								
3.	• the emotional attachment of owners to the active competition horse plays a less important role								
4.	• owners are better informed about possible diagnostics and therapies.								
5.	• owners have higher expectations of me and my medical services.								
6.	• treatment regression occurs more frequently due to poor owner compliance (e.g. training too early).								
7.	• my reputation plays a more important role.								
8.	• owners approach me more often with clear treatment ideas.								
9.	• owners show greater understanding of necessary diagnostics and/or treatments.								
10	• it is more burdensome to include interests of owners (e.g. sporting success) in veterinary decision-making processes.								
11	• owners of active competition horses communicate with each other more about veterinary activities.								
12	• situations occur more frequently in which the performance expectations of the animal owners are placed above the horse's welfare.								

2. *On average, how often does it happen that keepers of active show horses want a therapy that is not medically indicated but supposed to improve the performance?*
On average, how often did it happen that keepers of active show horses wanted a therapy that was not medically indicated but supposed to improve the performance?

- Never
- Less than once a month
- 1–2 times per month
- 3–4 times per month
- 5–10 times per month
- More than 10 times per month
- I don’t know

A. *To what extent do the following factors on the part of the animal keepers influence the wish for a therapy that goes beyond your veterinary recommendation?*
To what extent did the following factors on the part of the animal keepers influence the wish for a therapy that went beyond your veterinary recommendation?
1 = not at all; 2 = very little; 3 = moderately; 4 = relatively strongly; 5 = strongly und 6 = I don’t know

		1	2	3	4	5	6
1.	Wish to improve the animal’s performance						
2.	Belief that the treatment is in the animal’s best interest						
3.	Positive experience with their own animals or animals they know which got this treatment						
4.	Medical information they found on the internet						
5.	Second expert opinion						
6.	Advice by people from the field of equestrian sports (e.g. coach, other animal keepers)						
7.	Health insurance of the animal						

3. *On average, how often does it happen that keepers of active show horses refuse a therapy you recommend?*
On average, how often did it happen that keepers of active show horses refused a therapy you recommended?

- Never
- Less than once a month
- 1–2 times per month
- 3–4 times per month
- 5–10 times per month
- More than 10 times per month
- I don’t know

A. *To what extent do the following factors on the part of the animal keepers influence the refusal of a therapy you recommend?*
To what extent did the following factors on the part of the animal keepers influence the refusal of a therapy you recommended?
1 = not at all; 2 = very little; 3 = moderately; 4 = relatively strongly; 5 = strongly und 6 = I don’t know

		1	2	3	4	5	6
1.	Limited financial means						
2.	Belief that the treatment is not in the animal’s best interest						
3.	Negative experience with their own animals or animals they know which got this treatment						
4.	Animal keeper’s wish to get a second expert opinion						
5.	Dissuasion by people from the field of equestrian sports (e.g. coach, other animal keepers)						
6.	Due to the recommended therapy, the horse cannot be trained for a longer period of time.						

4. *When you think of your work at horse shows, how often are you faced with the following situations? (logical question → only if the answer to question 10 in block A is yes)*
When you think back of your work at horse shows, how often were you faced with the following situations?
1 = not at all; 2 = very little; 3 = moderately; 4 = often; 5 = very often und 6 = I don't know

		1	2	3	4	5	6
1.	In the warm-up arena, I observe riders and trainers using improper training methods.						
2.	Riders want to compete with their competition horses despite inadmissible medication.						
3.	Animal owner(s) presenting competition horses with low-grade lameness.						
4.	When examining the equipment, I come across unfairly prepared equipment (e.g. gaiters, fly ears).						
5.	When I point out a violation to riders, they show understanding.						
6.	Disagreements arise with competition organizers over the implementation of horse inspections.						
7.	There is a disagreement with the competition judges over the assessment of the health condition of the competition horse.						

Block C: Medical aspects when caring for active show horses

1. *How much of your practical work with active show horses is … (<10%, 10% to 100%, I don't know)*
How much of your practical work with active show horses was …

- preventive treatments (vaccinations, dental treatment etc.)
- emergency treatment
- primarily curative treatments (chronic diseases, lameness etc.)
- primarily performance-enhancing treatments

2. *When you think of the treatment of active show horses, to what extent do you agree with the following statements?*
When you think back of the treatment of active show horses, to what extent do you agree with the following statements?

1 = strongly disagree; 2 = disagree; 3 = somewhat disagree; 4 = neutral (neither agree nor disagree)); 5 = somewhat agree; 6 = agree, 7 = strongly agree and 8 = I don't know

		1	2	3	4	5	6	7	8
1.	Compared with leisure horses, active show horses more often present for preventive examinations.<br>Compared with leisure horses, active show horses more often presented for preventive examinations.								
2.	Keepers of active show horses use additional complementary services (e.g. osteopathy) more often.<br>Keepers of active show horses used additional complementary services (e.g. osteopathy) more often.								
3.	For active show horses, my diagnosis is more detailed.<br>For active show horses, my diagnosis was more detailed.								
4.	Keepers are more willing to agree to diagnostic measures in active show horses up to ten years than in older horses.<br>Keepers were more willing to agree to diagnostic measures in active show horses up to ten years than in older horses.								
5.	In principle, I think that performance-enhancing therapies for active show horses are justified.								
6.	In higher performance classes of the horses, the number of diseases increases.<br>In higher performance classes of the horses, the number of diseases increased.								
7.	In higher performance classes of the horses, the number of diseases decreases.<br>In higher performance classes of the horses, the number of diseases decreased.								

- *On average, how often does it happen that active show horses are presented to you only after the show season, although potential problems already occurred during the season?*
  On average, how often did it happen that active show horses were presented to you only after the show season, although potential problems had already occurred during the season?

- Never
- Less than once a month
- 1–2 times per month
- 3–4 times per month
- 5–10 times per month
- More than 10 times per month
- I don’t know

- *Training and husbandry systems are two important components in the field of equestrian sports.*
  In your opinion, how large is the share of diseases in active show horses that result from too intense and/or wrong training?

- None
- 1–5%
- 6–10%
- 11–20%
- 21–30%
- 31–40%
- 41–50%
- 51–60%
- 61–70%
- 71–80%
- 81–90%
- 91–100%
- I don’t know

- *In your opinion, how large is the share of diseases in active show horses that occur due to unsuitable husbandry systems?*

- None
- 1–5%
- 6–10%
- 11–20%
- 21–30%
- 31–40%
- 41–50%
- 51–60%
- 61–70%
- 71–80%
- 81–90%
- 91–100%

- I don’t know

- *Please estimate the share of diseases in active show horses that can be assigned to the respiratory tract, the locomotor system and the gastrointestinal tract. (<10 %, 10 % to 100 %, I don’t know)*

  ⇒ Respiratory tract
  ⇒ Locomotor system
  ⇒ Gastrointestinal tract

- *What are the **three most frequent** orthopaedic diseases in the active show horses you treat? Please tick all that apply.*
  *What were the **three most frequent** orthopaedic diseases in the active show horses you treated? Please tick all that apply.*

- Tendon injury at the front suspensory ligament
- Tendon injury at the back suspensory ligament
- Tendon injury at the superficial flexor tendon
- Tendon injury at the deep flexor tendon
- Annular ligament syndrome
- Palmar Foot Pain
- Kissing Spines
- Arthrosis coffin joint
- Arthrosis pastern joint
- Arthrosis fetlock
- Arthrosis of the facet joints
- Spavin
- Problems of the sacroiliac joint

- *On average, how often does it happen that animal keepers want joint injections only to improve the performance of the horse?*
  On average, how often did it happen that animal keepers wanted joint injections only to improve the performance of the horse?

- Never
- Less than once a month
- 1–2 times per month
- 3–4 times per month
- 5–10 times per month
- More than 10 times per month
- I don’t know

- *Which of the following preparations do you prefer for joint injections in active show horses? Please tick the preparations that apply.*
  Which of the following preparations did you prefer for joint injections in active show horses? Please tick the preparations that apply.

- Hyaluronic acid
- Triamcinolone
- Methylprednisolone acetate
- Betamethasone
- Glycosaminoglycans
- Polyacrylamide (e.g. Arthramid or similar)

- Bisphosphonates (e.g. Clodronate disodium (Osphos), Tiludronate disodium (Tildren))
- Platelet Rich Plasma
- Autologous conditioned plasma/serum (e.g. ACP, IRAP or similar)

Block D: case vignettes

In conclusion, we would like to present you four case vignettes that elucidate different aspects of show horse care.

CASE VIGNETTE 1:

A horse keeper wants to take part in a national jumping show with his active show horse in three weeks. The horse has already shown low-grade irregular rhythm several times at both forelegs and at the left hind leg. The cause has not been clarified due to the low-grade and transient lameness. Currently, the horse is free of lameness. To support the performance additionally to his training, the horse keeper wants you to inject into both coffin joints at the foreleg and the left talocrural joint. According to the keeper's information, his veterinarian has done that several times. He turns down your advice to take further diagnostic action.

What would you do?

- Treat the horse's joints according to the keeper's wish
- Not treat the horse's joints according to the keeper's wish
- Other

On answer 1:

Please move each of the following reasons into the category that reflects best how important this reason was for your decision. You can use the fields more than once.

- The injections into the joints do not do any harm to the horse.
- I help the horse to move without pain.
- I help the animal keeper to reach a better result in the show.
- Otherwise I risk that my client uses the services of a colleague.

Very important	Important	Less important	Not important at all	I don't know

On answer 2:

Please move each of the following reasons into the category that reflects best how important this reason was for your decision. You can use the fields more than once.

- In do not provide intraarticular treatment without a diagnostic examination.
- I do not do joint injections only to improve the performance.
- Currently, the horse does not show lameness.
- When I do the joint injections, the risk of possible complications is too high.

Very important	Important	Less important	Not important at all	I don't know

On answer 3:

Please indicate your next steps:

– ***Free text field –***

CASE VIGNETTE 2:

A desmopathy of the origin of the suspensory ligament at the left hind leg was diagnosed in an eleven-year-old gelding. As this horse is an active show horse with very good dressage pedigree and, thus, of high quality, the keeper would like to have the option to train the horse in her free time. The keeper asks you to do a neurectomy to enable leisure sports training free of pain for the horse.

What would you advise the animal keeper to do?

- I would advise her to do the surgery.
- I would advise her against the surgery.
- Other

On question 1:

Please move each of the following reasons into the category that reflects best how important this reason was for your decision. You can use the fields more than once.

- Enabling further training of the horse
- Concern that the keeper will see another veterinarian who will do the neurectomy
- The quality of life of the horse that has been an active show horse so far will be improved because movement free of pain will be guaranteed.

Very important	Important	Less important	Not important at all	I don't know

On answer 2:

Please move each of the following reasons into the category that reflects best how important this reason was for your decision. You can use the fields more than once.

- A neurectomy is not medically indicated.
- I am afraid that the keeper will take part in shows again after the neurectomy.
- Further training can sharpern the desmopathy of the origin of the suspensory ligament.
- I do not carry out treatments of this kind.

Very important	Important	Less important	Not important at all	I don't know

On answer 3:

Please indicate what you would advise the keeper to do:

– ***Free text field –***

CASE VIGNETTE 3:

A five-year-old horse that is active in jumping shows has a high-grade stridor after longer, exhausting training. Reduced performance cannot be found. The keeper is concerned.
After a clinical examination and further diagnosis, you diagnose a left laryngeal hemiplegia.

What would be your next step?

- I would recommend a surgery (laryngoplasty/ventricular cordectomy).
- At the moment, I would advise against a surgery (laryngoplasty/ventricular cordectomy).
- Other

On answer 1:

Please move each of the following reasons into the category that reflects best how important this reason was for your decision. You can use the fields more than once.

- The horse should be used as a show horse.
- To reduce/prevent further respiratory problems
- To ensure sufficient oxygen saturation of the horse and prevent exercise intolerance
- To improve the horse’s quality of life

Very important	Important	Less important	Not important at all	I don’t know

On answer 2:
Please move each of the following reasons into the category that reflects best how important this reason was for your decision. You can use the fields more than once.

- The active show horse does not show any signs of exercise intolerance.
- A laryngoplasty/ventricular cordectomy is not a suitable treatment of this patient.
- I treat the active show horse with a bronchodilator and try to solve the problem in this way.
- I do not carry out surgeries of this kind (no surgery facilities).

Very important	Important	Less important	Not important at all	I don’t know

On answer 3:
Please indicate your next steps:
– ***Free text field –***

CASE VIGNETTE 4:
You are one of three veterinarians at an international competition. During the VetCheck on the day before the competition you discover low-grade lameness in one of the dressage horses. Your colleagues are of the opinion that the animal is "fit for competition" according to the tournament regulations and that a start permit can be issued.

How would you proceed? (Block A, Frage 11a)

- I agree with the opinion of my colleagues and give a start permission.
- I am deciding against the start of the horse
- Other

On answer 1:
Please move each of the following reasons into the category that reflects best how important this reason was for your decision. You can use the fields more than once.

- It is a low-grade lameness and the horse is “fit enough to compete.”
- Excluding the rider carries reputational risk and may have negative consequences for me as a horse competition veterinarian.
- I feel well covered by the competition regulations, which simply state that the horse must be “fit enough to compete”.

Very important	Important	Less important	Not important at all	I don’t know

On answer 2:
Please move each of the following reasons into the category that reflects best how important this reason was for your decision. You can use the fields more than once.

- The welfare of the horse is my priority.
- To me, even a slight degree of lameness is a limitation of the animal's fitness to compete in this competition.
- The horse competition rules do not provide me with any safeguards if I were to decide to enter the horse in the competition.
- I would like to expose grievances without any exception at international competitions.

Very important	Important	Less important	Not important at all	I don't know

On answer 3:
Please indicate your next steps:
– ***Free text field –***

Thank you very much for participating in this survey.
